# Spatiotemporal Differences in Presentation of CD8 T Cell Epitopes during Hepatitis B Virus Infection

**DOI:** 10.1128/JVI.01457-18

**Published:** 2019-02-05

**Authors:** Atefeh Khakpoor, Yi Ni, Antony Chen, Zi Zong Ho, Vincent Oei, Ninghan Yang, Reshmi Giri, Jia Xin Chow, Anthony T. Tan, Patrick T. Kennedy, Mala Maini, Stephan Urban, Antonio Bertoletti

**Affiliations:** a Emerging Infectious Diseases Program, Duke-NUS Medical School, Singapore, Republic of Singapore; b Department of Infectious Diseases, Molecular Virology, University Hospital Heidelberg, Heidelberg, Baden-Württemberg, Germany; c German Center for Infection Research, Heidelberg, Germany; d Infectious Disease and Vaccines, Janssen Pharmaceuticals, Beerse, Belgium; e Lion TCR Private Limited, Singapore, Republic of Singapore; f Singapore Immunology Network, A*STAR, Singapore, Republic of Singapore; g Division of Infection and Immunity, University College London, London, United Kingdom; h Hepatology, Centre for Immunobiology, Blizard Institute, Barts and The London School of Medicine and Dentistry, Queen Mary University of London, London, United Kingdom; University of Southern California

**Keywords:** antigen presentation, CD8^+^ T cells, chronic hepatitis B patient, HBV DNA integration, HLA class I, liver, hepatitis B virus

## Abstract

Distinct populations of hepatocytes infected with hepatitis B virus (HBV) or only harboring HBV DNA integrations coexist within an HBV chronically infected liver. These hepatocytes express HBV antigens at different levels and with different intracellular localizations, but it is not known whether this heterogeneity of viral antigen expression could result in an uneven hepatic presentation of distinct HBV epitopes/HLA class I complexes triggering different levels of activation of HBV-specific CD8^+^ T cells. Using antibodies specific to two distinct HLA-A*02:01/HBV epitope complexes of HBV nucleocapsid and envelope proteins, we mapped their topological distributions in liver biopsy specimens of two anti-hepatitis B e antigen-positive (HBe^+^) chronic HBV (CHB) patients. We demonstrated that the core and envelope CD8^+^ T cell epitopes were not uniformly distributed in the liver parenchyma but preferentially located in distinct and sometimes mutually exclusive hepatic zones. The efficiency of HBV epitope presentation was then tested *in vitro* utilizing HLA-A*02:01/HBV epitope-specific antibodies and the corresponding CD8^+^ T cells in primary human hepatocyte and hepatoma cell lines either infected with HBV or harboring HBV DNA integration. We confirmed the existence of a marked variability in the efficiency of HLA class I/HBV epitope presentation among the different targets that was influenced by the presence of gamma interferon (IFN-γ) and availability of newly translated viral antigens. In conclusion, HBV antigen presentation can be heterogeneous within an HBV-infected liver. As a consequence, CD8^+^ T cells of different HBV specificities might have different antiviral efficacies.

**IMPORTANCE** The inability of patients with chronic HBV infection to clear HBV is associated with defective HBV-specific CD8^+^ T cells. Hence, the majority of immunotherapy developments focus on HBV-specific T cell function restoration. However, knowledge of whether distinct HBV-specific T cells can equally target all the HBV-infected hepatocytes of a chronically infected liver is lacking. In this work, analysis of CHB patient liver parenchyma and *in vitro* HBV infection models shows a nonuniform distribution of HBV CD8^+^ T cell epitopes that is influenced by the presence of IFN-γ and availability of newly translated viral antigens. These results suggest that CD8^+^ T cells recognizing different HBV epitopes can be necessary for efficient immune therapeutic control of chronic HBV infection.

## Abstract (precis)

The inability of patients with chronic HBV infection to clear HBV is associated with defective HBV-specific CD8^+^ T cells. Hence, the majority of immunotherapy developments focus on HBV-specific T cell function restoration. However, knowledge of whether distinct HBV-specific T cells can equally target all the HBV-infected hepatocytes of a chronically infected liver is lacking. In this work, analysis of CHB patient liver parenchyma and *in vitro* HBV infection models shows a nonuniform distribution of HBV CD8^+^ T cell epitopes that is influenced by the presence of IFN-γ and availability of newly translated viral antigens. These results suggest that CD8^+^ T cells recognizing different HBV epitopes can be necessary for efficient immune therapeutic control of chronic HBV infection.

## INTRODUCTION

CD8^+^ T cells play an important role in protecting the host against viral infections. Using specific T cell receptors (TCR), CD8^+^ T cells recognize and subsequently lyse virus-infected cells expressing HLA class I/viral peptide complexes on their surfaces (1). The efficiency of HLA class I/viral peptide complex formation is essential for the recognition of virus-infected cells by CD8^+^ T cells (2); viruses that can establish chronic infection such as human cytomegalovirus (HCMV) and HIV have evolved strategies to modulate either processing or presentation of these complexes (3).

The ability of CD8^+^ T cells to recognize hepatitis B virus (HBV)-infected hepatocytes has been studied in chimpanzees (4) and humanized chimeric mouse models (5). However, due to the technical difficulties in establishing HBV infection in primary human hepatocytes (PHH) *in vitro* (6), the efficiency of HBV epitope presentation after infection has never been analyzed in detail. Most studies on CD8^+^ T cell recognition of HBV-infected targets have employed experimental systems in which HBV antigen expression was driven by either viral vector transfections (Ebola virus, vaccinia virus, or adenovirus) (7–9) or HBV DNA integration into the host genome (HepG2.2.15 or HBV transgenic mice) (10–12). Only following the recent characterization of the HBV entry receptor human sodium taurocholate cotransporting polypeptide (hNTCP) (13) has a robust HBV infection system been established in HepG2-hNTCP-A3 cells (14) allowing the study of human HBV core-specific CD8^+^ T cell recognition of HBV-infected targets *in vitro* (15). However, whether distinctive epitopes originating from different HBV proteins are differently presented during infection is not known. Equally, the ability of HepG2-hNTCP-A3 cells to process and present viral antigens may differ from that of normal hepatocytes since defects in antigen presentation have been suggested to occur in hepatocellular carcinoma (HCC) cells (16).

Similarly, although HLA class I/HBV peptide complexes can be directly visualized on liver biopsy specimens of chronically infected patients (17, 18), knowledge related to the efficiency and kinetics of the generation of HLA class I/HBV peptide complexes in chronic HBV (CHB)-infected livers is limited (19, 20). Studies investigating the localization of HBV-infected hepatocytes in the liver of patients with chronic hepatitis B showed a complex mosaic of cells expressing HBV antigens at different levels and localizations (21, 22) and with broad differences in the ratio between HBV surface antigen (HBsAg) and covalently closed circular DNA (cccDNA) levels (23–25). This differential antigenic expression is likely caused by the concomitant presence of hepatocytes infected with HBV for different durations and/or the production of HBV antigens from either integrated HBV DNA or cccDNA (25, 26).

Overall, whether HBV-specific CD8^+^ T cells are able to distinguish distinct populations of HBV antigen-expressing hepatocytes is unknown. Investigations of HBV-specific T cells during natural infection have focused exclusively on their quantity (7, 27, 28), function (29), and localization (28, 30), while the ability of hepatocytes to present HBV epitopes to their cognate HBV-specific CD8^+^ T cells has been neglected. To fill this knowledge gap, we first utilized T cell receptor-like antibodies (TCRL-Abs) specific for two distinct HBV epitopes derived from envelope and nucleocapsid antigen and presented by HLA-A*02:01 to analyze their distribution in the liver of CHB patients.

We then compared the *in vitro* efficiency of presentation of different HLA class I/HBV epitopes in HBV-infected PHH and in hepatocyte-like cell lines (HepG2-hNTCP-A3, HepG2.2.15, HepG2-Env, and PLC/PRF5/HLA-A2^+^) infected by HBV or expressing HBV antigens from HBV DNA integration. We demonstrated that distinct epitopes are presented with differing efficiencies and that the presence of gamma interferon (IFN-γ) and availability of newly translated viral antigens modulate the quantity of HBV epitope presentation.

## RESULTS

### Heterogeneous distribution of CD8^+^ T cell core and envelope epitopes in chronically HBV-infected human liver.

We first performed a comparative analysis of the distribution of two HBV epitope/HLA class I complexes within HBV-infected livers. We utilized antibodies that have already been demonstrated to specifically recognize the HLA-A*02:01/HBc18-27 (defined as Ab A2-HBc18) and the HLA-A*02:01/HBs183-191 (defined as Ab A2-HBs183) complexes in HBV-infected cells and in biopsy specimens of HLA-A*0201-positive (HLA-A*0201^+^) patients with CHB (17, 18).

**FIG 1:**
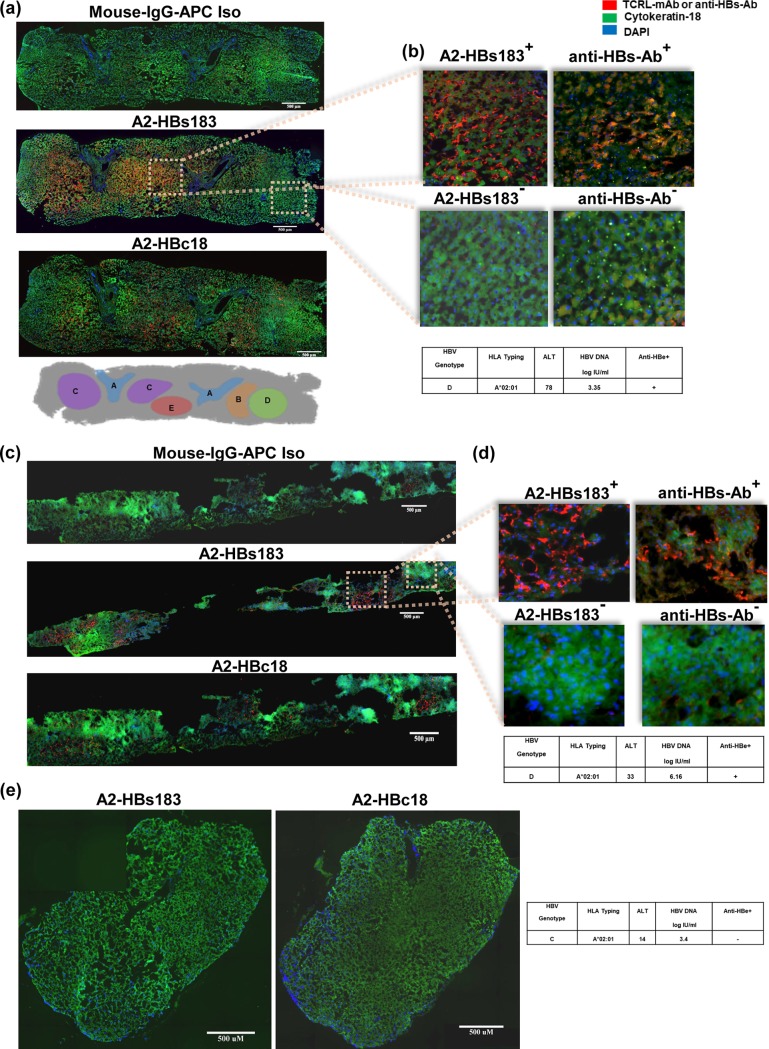
Spatial distribution of HLA/HBV epitope complexes in HBV chronically infected livers. Comparative analysis of CHB patient liver biopsy specimens demonstrated a nonuniform distribution of HBV epitope presentation in liver parenchyma. (a to d) Consecutive sections of two CHB patient liver biopsy specimens (specimens 1 and 2 in Table 1), stained with isotype (Iso) control antibody (anti mouse IgG-APC) and TCR-like antibody specific for HLA-A*02:01/HBs183-91 (A2-HBs183) and for HLA-A*02:01/HBc18-27 (A2-HBc18) complexes and with anti-HBs antibody (insets b and d). Regions positive for antibody staining (TCR-like and anti-HBs antibodies) are in red, hepatocytes are stained with cytokeratin 18 in green, and nuclei of cells are stained with DAPI in blue. The images were captured using TissueFAXS immunofluorescent microscopy. A schematic representation of the distribution of the two HLA-A*02:01/HBV epitope complexes in hepatic parenchyma of patient 1 is shown in panel a. Region A marks the fibrotic portal region. Region B indicates parts only positive for A2-HBc18 complexes while region C is positive for A2-HBs183 complexes. Regions D and E, respectively, are negative and positive for both HLA/HBV epitope complexes. Region E is positive for both A2-HBc18 and A2-HBs183 complexes. (e) Representative liver biopsy specimen images of an HLA-A*02:01-positive patient infected with HBV genotype C (specimen 4 in Table 1), stained with TCR-like antibodies. Inserted tables summarize the clinical and virological features of each patient.

Liver biopsy specimens of 8 HLA-A*0201^+^ CHB patients (Table 1) were stained with the above-mentioned antibodies and analyzed with TissueFAXS immunofluorescent microscopy to create high-resolution images of whole biopsy specimens. Note that since both Ab A2-HBc18 and Ab A2-HBs183 antibodies are raised in mouse and not directly conjugated with fluorochrome, this comparative analysis of the different localizations of the A2-HBc18 and A2-HBs183 complexes had to be done by staining individual tissue slides corresponding to two consecutive sections. Table 1 shows that only 3 out of 8 HLA-A*02:01^+^ liver biopsy specimens showed positive staining. Interestingly, all three positive biopsy specimens are from CHB patients of Caucasian ethnicity who are infected with genotype D (Fig. 1a to d), while five of the negative biopsy specimens were derived from CHB patients of Chinese ethnicity infected with genotypes B and C (Fig. 1e). This is likely to be caused by the natural amino acid substitutions present within the HBc18-27 and HBs183-91 sequences present in HBV genotypes B and C while both antibodies were raised utilizing epitope sequences of HBV genotype D (31).

**TABLE 1:** Virological and clinical characteristics of CHB patient liver biopsy specimens^*a*^

Biopsy specimen no.^*b*^	HBV genotype	TCRL mAb-APC
1	D	Positive
2	D	Positive
3	D	Positive
4	C	Negative
5	C	Negative
6	C	Negative
7	B	Negative
8	B	Negative

a All patient specimens were HLA type A*02:01 and positive for HBsAg and HBeAg.

b Specimens are shown in figures as follows: specimen 1, Fig. 1a and b; specimen 2, Fig. 1c and d; specimen 4, Fig. 1e.

The staining of the two antibodies was not uniformly distributed among the hepatic parenchyma but varied in intensity and localization. Figure 1a and c show a representative image of two CHB patients positive for anti-hepatitis B e antigen (anti-HBe^+^). A2-HBc18 and A2-HBs183 complexes were visualized only in the hepatic parenchyma (Fig. 1a, c) and not in the fibrotic portal tracts (Fig. 1a and c), further confirming the specificity of our antibodies. Furthermore, not only was there a nonuniform distribution of both epitopes within the hepatic tissue, but also the two different HLA class I/HBV epitopes can be detected in distinct anatomical regions. For example, in region B (according to the schematic in Fig. 1a), there was a robust detection of A2-HBc18 complexes, whereas A2-HBs183 complex detection was negligible (Fig. 1a). On the other hand, region C had predominant expression of A2-HBs183 complexes, with low or absent A2-HBc18 complex detection (Fig. 1a). Analysis of HBV antigen (HBV core antigen [HBcAg] and HBsAg) expression was performed in these two CHB patients. Figure 1b and d show that the regions of higher A2-HBs183 complex detection were topologically correlated with HBsAg expression. Unfortunately, technical problems hampered detection of HBcAg localization in these two biopsy specimens, preventing the parallel analysis of A2-HBc18 and HBcAg expression.

Finally, detection of these two HLA-A*02:01/HBV epitopes was completely negative in other hepatic parenchymal regions (region D). Similar results were observed in the biopsy specimen of a second CHB patient (anti-HBe^+^) which stained positive with both antibodies (Fig. 1c and d). Therefore, this analysis shows that, at least in anti-HBe^+^ CHB patients, expression of distinct HBV epitopes has a mosaic pattern of distribution.

### Establishing an *in vitro* system of HBV infection.

**FIG 2:**
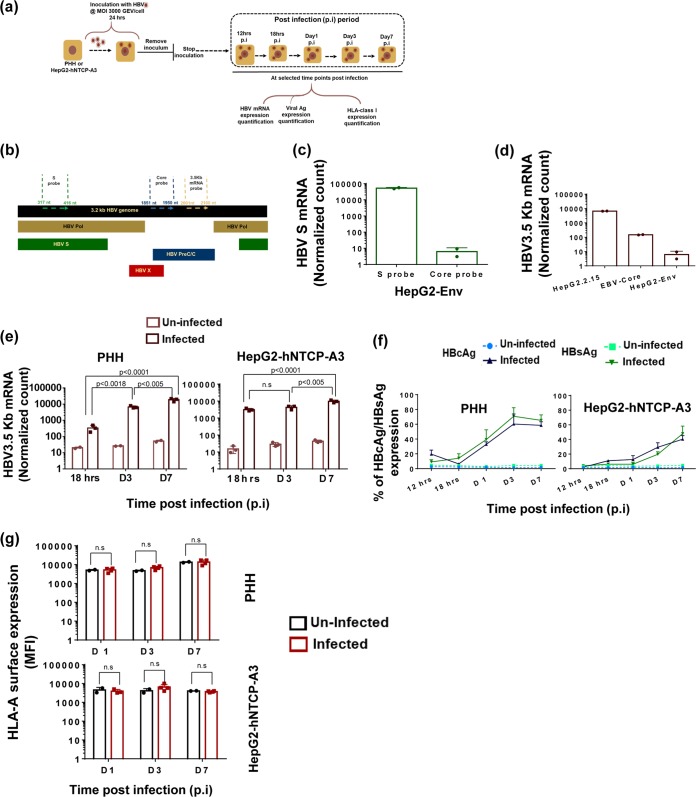
Establishment of *in vitro* HBV infection in PHH and HepG2-hNTCP-A3 cells. (a) Schematic representation of the experimental procedure utilized to infect HepG2-hNTCP-A3 cells and PHH. Cells were inoculated at a multiplicity of infection of 3,000 GEV/cell for 24 h. Cells were utilized for virological and immunological assays at the indicated times after removal of the inoculum (time 0) referred to as time postinfection. (b) NanoString probes specific to HBV 3.5-kb mRNA (pgRNA), HBV large S, and HBV core mRNA were designed. The map represents the regions on the HBV genome which are covered by each probe. (c and d) Probe specificities were tested in cells with overexpression of individual HBV proteins or full HBV DNA integration, as indicated (Table 2). Bars show normalized counts for the indicated mRNA obtained by NanoString technology in each cell line. The highest count in each cell line belongs to the probe more specific to the region of the HBV protein which the different cell lines are overexpressing. (c) HepG2-Env cell lines shows higher counts for the probe specific to a region of large S. (d) Highest counts of the probe specific to HBV 3.5-kb mRNA (pgRNA) was observed in HepG2.2.15 cells, which have active HBV replication. (e) HBV 3.5-kb mRNA expression in PHH and HepG2-hNTCP-A3 infected cells at the indicated durations of infection (D, days). Bars represent the normalized counts of HBV 3.5-kb mRNA (pgRNA) obtained using NanoString technology. The indicated *P* values represent the significant increase of viral replication over the time of infection (mean of 3 replicates). (f) Frequency of HBV-infected or uninfected cells in PHH and HepG2-hNTCP-A3 cells. Cells expressing HBcAg (blue) or HBsAg (green) at 12 and 18 h and at days 1, 3, and 7 postinfection are measured with anti-HBs- and anti-HBc-specific antibodies by flow cytometry analysis. A gradual increase in the frequency of HBcAg/HBsAg-positive cells is observed in both PHH and HepG2-hNTCP-A3 cells over infection time. (g) HLA-class I surface expression in HBV-infected PHH and HepG2-hNTCP-A3 cells measured using flow cytometry. The surface expression of HLA class I is compared to that of uninfected target cells over time (days 1 to 7).

In order to study the regulation of HBV-derived epitope presentation in a more controlled *in vitro* system, we established an infection system to mimic acute HBV infection (arbitrarily defined as events occurring 12 h to 7 days postinfection [p.i.]) using PHH and HepG2-hNTCP-A3 cells (Fig. 2a) The HLA class I compatibility between the target and our reagents (HLA-A*02:01-restricted HBc18-27 and HBs183-91-specific CD8^+^ T cell clones) and the two TCR-like antibodies (Ab-A2-HBc18 and Ab-A2-HBs183) was retained by using HLA-A*02:01^+^ PHH while HepG2-hNTCP-A3 cells are HLA-A*02:01^+^. Both PHH and HepG2-hNTCP-A3 cells were infected at a multiplicity of infection of 3,000 genome equivalents (GEV)/cell, as shown in the schematic in Fig. 2a. Establishment of productive HBV replication upon infection was confirmed by measuring HBV 3.5-kb mRNA (pregenomic RNA [pgRNA]) using NanoString technology while expression of HBcAg and HBsAg was quantified by flow cytometry using anti-HBs- and anti-HBc-specific antibodies at 12 h, 18 h, and days 1, 3, and 7 p.i. NanoString probe design is shown in Fig. 2b. The specificity of probes (results related to HBV S protein mRNA and HBV 3.5-kb mRNA are shown) was tested in cells overexpressing an individual HBV peptide (HepG2-Env) or harboring the full HBV genome (HepG2.2.15), as shown in Fig. 2c and d.

In both PHH and HepG2-hNTCP-A3 cells (Fig. 2e), pregenomic RNA was already detectable at 18 h p.i. and progressively increased until day 7. HBcAg and HBsAg detection also increased gradually from 18 h to day 7 p.i. in both PHH and HepG2-hNTCP-A3 cells. The frequency of cells positive for HBV antigens in HBV-infected cells was higher in hepatocytes (>60% cells positive for HBV antigens at day 3 p.i.) than in HepG2-hNTCP-A3 cells (∼40 to 50% at day 7 p.i.) (Fig. 2f). Interestingly, the frequency of HBsAg-expressing hepatocytes was slightly higher than that of core antigen-expressing cells, while this trend was opposite in HBV-infected HepG2-hNTCP-A3 cells.

**FIG 3:**
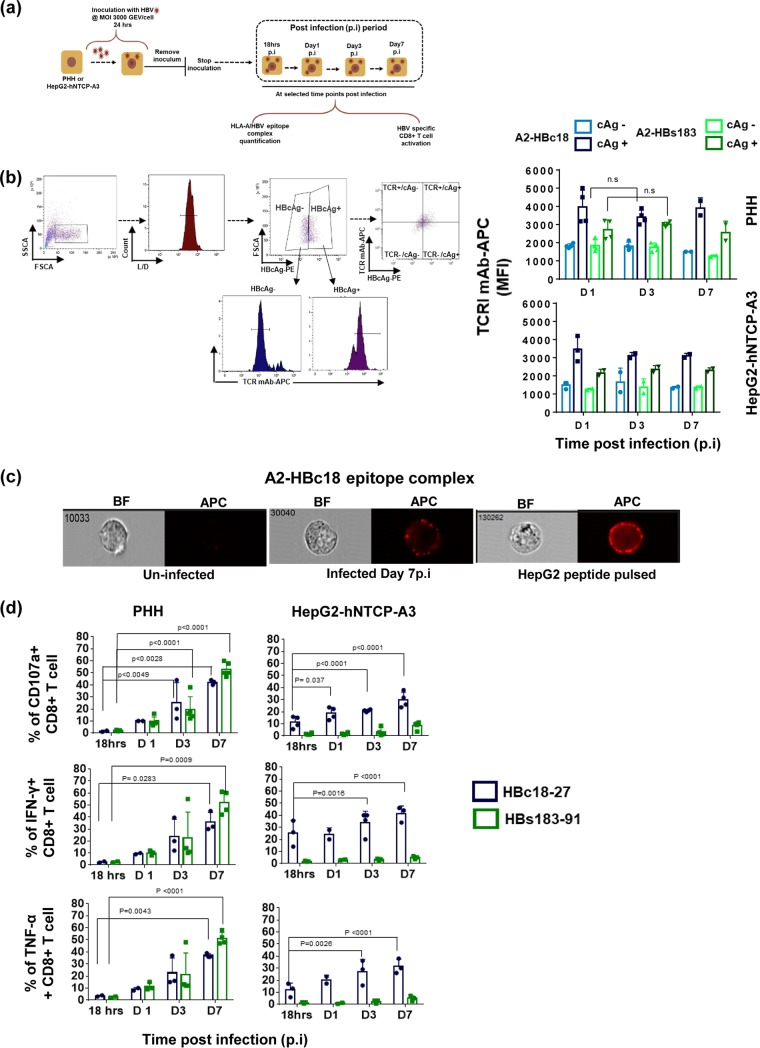
HLA/HBV epitope complex presentation on HBV-infected PHH and HepG2-hNTCP-A3 cells. (a) Schematic representation of HBV-infected PHH or HepG2-hNTCP-A3 cells utilized for immunological assays. (b) Quantity of HLA-A*02:01/HBV epitope complexes on the HBcAg-negative and -positive HBV-infected cells was measured using two TCR-like mAbs, A2-HBc18 and A2-HBs183, by flow cytometry analysis. The gating strategy is shown at left. Histograms display representative MFI values of TCR-like antibodies measured on gated cells positive or negative for HBcAg staining (using anti-HBc antibody) (L/D, Live/dead). The representative dot plot on the right shows the staining profile of a population positive for both HBcAg (*x* axis) and TCR-like mAb (*y* axis). On the right, bars show MFI values of A2-HBc18 and A2-HBs183 on HBcAg^+^ cells in comparison with the HBcAg^−^ population in both PHH and HepG2-hNTCP-A3 infected cells. Dots represents individual experiments. At least two replicates for indicated time points were performed. SSCA, side scatter, area; FSCA, forward scatter, area. (c) The surface distribution of A2-HBc18 is shown on HepG2-hNTCP-A3 cells infected at 7 days postinfection in comparison with HepG2 cells pulsed with 1 μg/ml of HBc18-27 peptide, using ImageStream analyzer. Representative images of cells show clustered distribution of complexes on infected targets and peptide-pulsed cells. Uninfected cells show negative background staining. BF, bright field; APC, allophycocyanin (fluorescent field). (d) Ability of HBc18-27- and HBs183-91-specific CD8^+^ T cells to recognize PHH and HepG2-hNTCP-A3 cells infected with HBV for the indicated times. Bars show the frequency of CD107a-expressing CD8^+^ T cells and activated IFN-γ- or TNF-α-positive CD8^+^ T cells among total CD8^+^ T cells after being cocultured for 5 h at an E/T ratio of 1:2. All data shown are the means ± standard deviations of at least three independent experiments.

Having established two *in vitro* HBV infection systems, we analyzed the hierarchy of HBV epitope presentation in both HBV-infected hepatocytes and HepG2-hNTCP-A3 cells (Fig. 3a, schematic). First, we tested whether the level of HLA class I expression was modified by HBV infection. In line with other evidence showing that HBV can replicate within hepatocytes without being sensed by innate immunity sensors (32), we did not detect variations in either HLA class I mRNA (data not shown) or protein expression levels upon infection in both systems (Fig. 2g).

We then analyzed the surface expression of HLA-A*02:01/HBV epitope complexes. HBV-infected hepatocytes and HepG2-hNTCP-A3 cells were stained with anti-HBcAg antibodies and with the two TCR-like antibodies (17) over the duration of infection. The expression of two HBV epitopes was analyzed after gating for cells which were productively synthesizing HBcAg (the gating strategy is shown in Fig. 3b). The quantity of the core- and envelope-derived epitopes did not increase in HBcAg-expressing targets over time. However, the presentation of both core and envelope epitopes was more efficient in PHH than in HepG2-hNTCP-A3 cells, and the A2-HBc18 complexes were presented more efficiently than the A2-HBs183 complexes in both hepatocytes and HepG2-hNTCP-A3 cells. In contrast to the distribution on peptide-pulsed targets (HepG2-pulsed with 1 μM peptide), the surface distribution of the HLA/HBV epitope complex on HBV-infected HepG2-hNTCP-A3 cells was in a discrete cluster (Fig. 3c).

We then tested the ability of HBc18-27- and HBs183-91-specific CD8^+^ T cells to recognize HBV-infected targets. By day 1 after HBV infection, hepatocytes activated both HBc18-27- and HBs183-91-specific CD8^+^ T cells (Fig. 3d). CD8^+^ T cell activation (tested as CD107a and IFN-γ/tumor necrosis factor alpha [TNF-α] expression in the experiments shown in Fig. 3d) progressively increased from day 1 to day 7, likely as a result of the increased quantity of hepatocytes expressing the two different epitopes. Furthermore, HBs183-91 CD8^+^ T cells were activated more efficiently by PHH than by HepG2-hNTCP-A3 infected cells (Fig. 3d), in line with the superior presentation ability of PHH detected by TCR-like antibody staining.

### Effect of IFN-γ on HBV CD8^+^ T cell epitope presentation.

**FIG 4:**
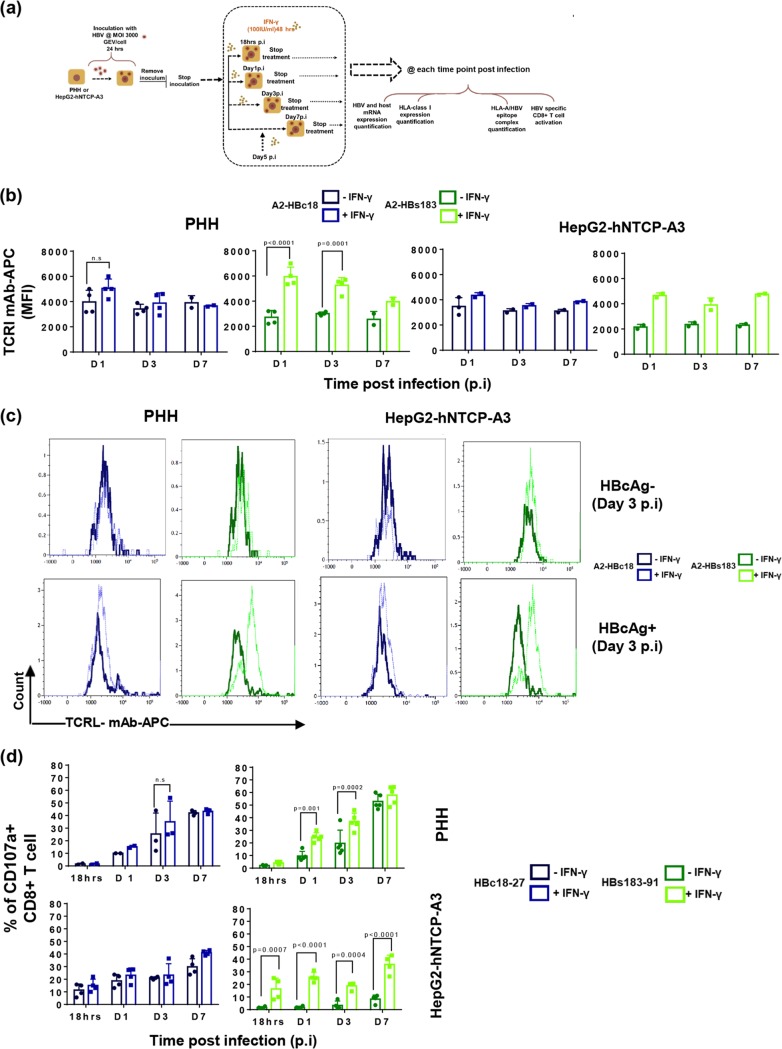
The effect of IFN-γ on HBV-epitope presentation. (a) Schematic of experimental procedure of IFN-γ pulsing (100 IU/ml, 48 hours) of HBV-infected HepG2-hNTCP-A3 cells and PHH. (b) Direct quantification of HLA-class I/HBV epitope complexes with Abs specific for A2-HBc18 and A2-HBs183 complexes in HBV-infected PHH or HepG2-hNTCP-A3 cells with or without IFN-γ. Bars show MFI values of HBcAg^+^ populations in untreated infected cells (dark shades) compared with the population in IFN-γ treated cells (bright shades). (c) Representative histogram plots of day 3 postinfection showing MFI values of A2-HBc18 and A2-HBs183 complexes on cell surface of HBcAg^−^ and HBcAg^+^ cells in infected PHH or HepG2-hNTCP-A3 cells. In each histogram the MFI value of the TCR-like mAb is shown in the presence (dotted lines) or absence (solid lines) of IFN-γ treatment. (d) Ability of CD8^+^ T cells specific for HBc18-27 and HBs183-91 to recognize infected PHH or HepG2-hNTCP-A3 cells for the indicated duration, with (bright shades) or without (dark shade) IFN-γ treatment (100 IU/ml, 48 h). Bars represent frequency of CD8^+^ T cells expressing CD107a among total CD8^+^ T cells cocultured with PHH and HepG2-hNTCP-A3 cells for 5 h at an E/T ratio of 1:2. All data shown are the means ± standard deviations of at least three independent experiments.

Efficient generation of viral epitopes in infected cells can be modulated by the presence of cytokines and particularly of IFN-γ, which is known to activate cellular immunoproteasomes (33). We tested the effect of IFN-γ treatment (at 100 IU/ml for 48 h) on HBV-infected hepatocytes and HepG2-hNTCP-A3 cells (Fig. 4a, schematic). As expected (33), IFN-γ treatment increased the surface expression of HLA class I molecules, as well as the mRNA expression level of the immunoproteasome subunit, PSMB8 and PSMB9 (data not shown).

We then directly measured the quantity of HLA-A*02:01/HBV epitope complexes with TCR-like antibodies in HBcAg^+^ and HBcAg-negative (HBcAg^−^) populations of infected targets (Fig. 4b). Note that treatment of IFN-γ did not have any effect on TCR-like antibody staining in the HBcAg-negative population in comparison to that with the HBcAg^+^ population of the infected targets (Fig. 4c). In contrast, in HBcAg^+^ populations, IFN-γ treatment increased the surface expression of A2-HBs183 complexes at all time points, but it had a more limited effect on A2-HBc18 expression (Fig. 4b). We then tested the impact of IFN-γ treatment on HBc18-27- and HBs183-91-specific CD8^+^ T cell recognition of infected targets. IFN-γ treatment did not alter HBc18-27-specific CD8^+^ T cell activation (Fig. 4d, left panels) but significantly increased HBs183-91-specific CD8^+^ T cell activation (Fig. 4d, right panels) (shown as CD107a^+^ CD8^+^ T cells) as early as day 1 postinfection, with approximately >40% of activated HBs183-91 CD8^+^ T cells detected by day 7 postinfection (Fig. 4d, right panels). Activation of CD8^+^ T cells measured by IFN-γ production displayed a similar pattern (data not shown). Thus, the presence of inflammatory cytokines (IFN-γ) affects epitope presentation in HBV-infected cells.

### HBV-epitope/HLA-A*02:01 complex presentation requires NTCP-mediated HBV internalization and synthesis of viral proteins.

**FIG 5:**
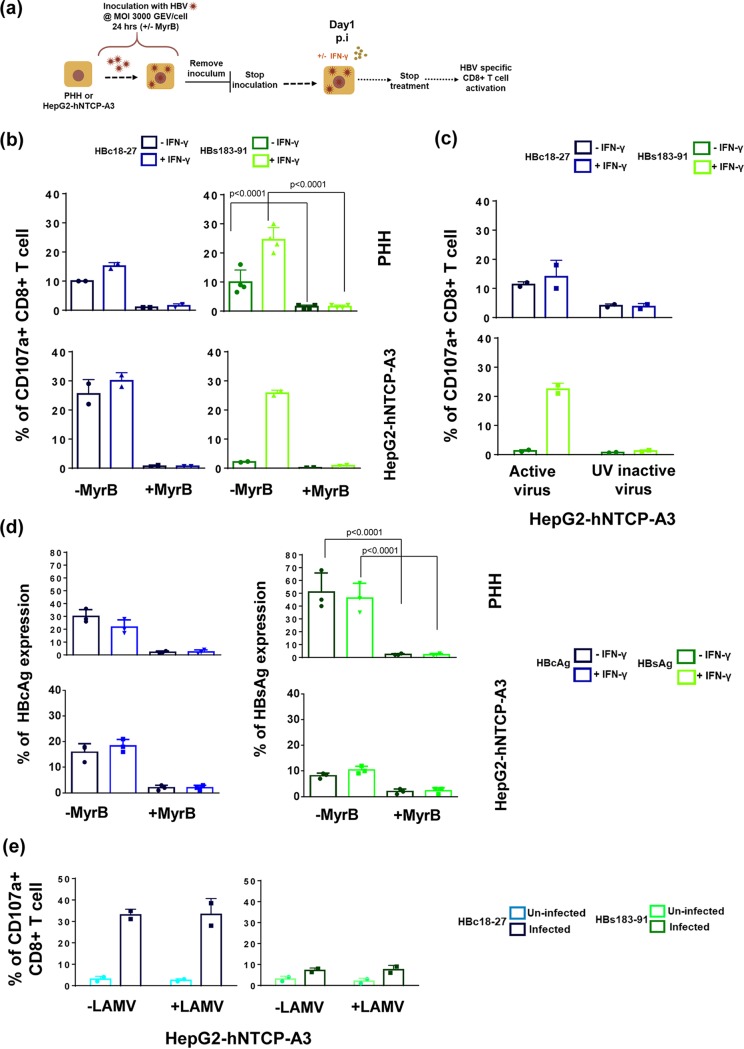
HBV epitope presentation requires NTCP-mediated infection and viral protein synthesis. (a) Schematic representation of infection of HepG2-hNTCP-A3 cells and PHH in the presence or absence of 800 nM Myrcludex B (MyrB). (b) Frequency of HBV-specific CD8^+^ T cells expressing CD107a among total CD8^+^ T cells after 5 h of coculture (E/T ratio of 1:2) with infected PHH or HepG2-hNTCP-A3 cells at day 1 postinfection. Targets were infected in the presence or absence of MyrB. In addition, cells were either treated with IFN-γ or left untreated as described in the legend of Fig. 4. (c) Ability of CD8^+^ T cells specific for HBc18-27 or HBs183-91 to recognize HepG2-hNTCP-A3 cells infected with HBV or UV-inactivated HBV in the presence or absence of IFN-γ. Bars show CD8^+^ T cells positive for CD107a among total CD8^+^ T cells after 5 h of coculture with target cells at day 1 postinfection (E/T ratio of 1:2). Data shown as means ± standard deviations of two independent experiments. (d) Expression of HBcAg and HBsAg in the presence or absence of MyrB treatment in both PHH and HepG2-hNTCP-A3 infected targets at day 1 p.i. is shown. Targets were either treated with IFN-γ (bright shades) or untreated (dark shades). (e) Frequency of CD107a-positive CD8^+^ T cells specific for HBc18-27 and HBs183-91, cocultured with HBV infected (dark shades) or uninfected (bright shades) HepG2-hNTCP-A3 cells treated or untreated with 10 μM lamivudine. Data are from day 3 postinfection.

It was previously shown that HBsAg can be efficiently cross-presented by dendritic cells and monocytes treated with inflammatory cytokines (34). Since HBV infection of PHH and HepG2-hNTCP-A3 cells was performed utilizing a high dose of virus (3,000 GEV/cell), we sought to determine if HBV antigen presentation by HLA class I molecules was the result of cross-presentation of exogenous viral antigens or of the processing of endogenously synthesized antigen. Both PHH and HepG2-hNTCP-A3 cells were infected with HBV in either the presence or absence of the viral entry inhibitor Myrcludex B (MyrB) peptide (800 nM) with or without IFN-γ treatment (Fig. 5a). In both infection systems HBs183-91- and HBc18-27-specific CD8^+^ T cell activation was significantly reduced (Fig. 5b).

Furthermore, HBV infection was carried out with UV-inactivated virus. Due to the limited number of PHH, this experiment was performed only with HepG2-hNTCP-A3 cells. Regardless of IFN-γ treatment, HBs183-91- and HBc18-27-specific CD8^+^ T cell activation was significantly reduced in UV-inactivated HBV-infected targets (Fig. 5c). Thus, these results show that the generation of HBs183-91 and HBc18-27 epitopes is not the result of cross-presentation of HBV proteins present in the initial HBV inoculum. On the contrary, since both MyrB (Fig. 5d) and UV inactivation (data not shown) suppress HBV antigen expression, epitope presentation requires HBV entry and synthesis of viral proteins. Note that experiments performed with HBV-infected HepG2-hNTCP-A3 cells treated with a nucleoside analogue (NA) (lamivudine; 10 μM) did not suppress HBV epitope presentation (Fig. 5e) since NA blocks HBV DNA and not protein synthesis.

### CD8^+^ T cell recognition of targets with HBV DNA integration.

A variable quantity of hepatocytes present in chronically infected livers are not HBV infected but carry HBV DNA integrations, and this phenomenon is more intense in anti-HBe^+^ patients (23, 25, 26). We analyzed HLA class I/HBV epitope complex expression on target cells with HBV DNA integration: HepG2.2.15 (HepG2 cells with full HBV genome integration) (10), HepG2-Env (HepG2 cells with full HBV genotype D envelope) (35), and PLC/PRF5/HLA-A2^+^ (natural HCC line with partial HBV surface antigen DNA integration [36] transduced with the HLA-A*02:01 molecule) cells.

**FIG 6:**
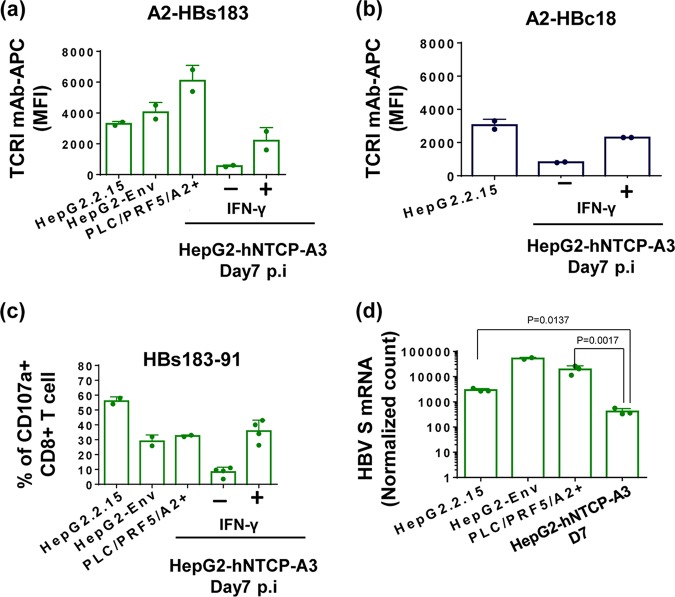
Superior HBV CD8^+^ T cell epitope presentation on targets producing HBV antigens from HBV DNA integration. (a) Direct quantification of A2-HBs183 complexes on the surface of hepatoma cells with HBV DNA integration (HepG2.2.15, HepG2-Env, and PLC/PRF5-A2^+^) in comparison with infected HepG2-hNTCP-A3 cells at day 7 postinfection, treated or untreated with IFN-γ. Cells were stained with A2-HBs183 antibody as indicated previously. Bars show MFI value of the indicated antibody measured by an ImageStream analyzer. (b) Bars show MFI values of A2-HBc18 complexes on the surface of cells with full HBV genome integration, HepG2.2.15 cells, in comparison with infected HepG2-hNTCP-A3 cells at day 7 postinfection in the presence or absence of IFN-γ. MFI values were measured using an ImageStream analyzer. (c) Ability of HBs183-91-specific CD8^+^ T cells to recognize target cells with HBV DNA integration (HepG2.2.15, HepG2-Env, and PLC/PRF5-A2^+^) and HBV-infected HepG2-hNTCP-A3 cells (day 7 postinfection with or without IFN-γ treatment). Bars represent activation of CD8^+^ T cells measured through CD107a expression. (d) Expression of HBV large S mRNA was quantified on target cells with HBV DNA integration (HepG2.2.15, HepG2-Env, and PLC/PRF5-A2^+^). Bars represent the numeric normalized count of mRNA measured by NanoString technology. These values are compared to similar quantifications in HBV-infected HepG2-hNTCP-A3 target cells at day 7 postinfection.

All cell lines produced HBsAg constitutively and showed higher expression of A2-HBs183 complexes than HBV-infected HepG2-hNTCP-A3 cells at day 7 postinfection (Fig. 6a). The quantity of complexes was higher than that observed in infected HepG2-hNTCP-A3 cells in the presence of IFN-γ. Furthermore, cells with full HBV genome integration (HepG2.2.15) could present A2-HBc18 complexes at a higher level than that quantified in HBV-infected HepG2-hNTCP-A3 cells, regardless of the presence of IFN-γ (Fig. 6b).

Moreover, these cells could activate HBs183-91 CD8^+^ T cells as efficiently as, or even better than, HepG2-hNTCP-A3-infected targets in the presence of IFN-γ (Fig. 6c). Since viral epitopes should be derived preferentially from newly synthetized proteins, we analyzed whether the increased quantity of HBV epitopes derived from the protein coded by integrated HBV DNA was proportional to the level of mRNA. We quantified HBV large S protein mRNA expression levels in the cell lines containing HBV DNA integration in comparison with levels in acutely infected HepG2-hNTCP-A3 targets. The level of HBs mRNA in cells with HBV DNA integration was higher (Fig. 6d). Taken together, these data show that at least in HepG2-derived lines, HBV epitopes are presented in higher quantities in targets producing antigens from HBV DNA integration. The epitope presentation is proportional to the quantity of HBV antigen mRNA detected in the different targets, suggesting that the quantity of newly synthetized proteins might regulate the efficient presentation of HBV peptides.

### Differential final intracellular distribution of HBV antigens does not alter HBV epitope presentation.

HepG2-hNTCP-A3 lines could be maintained *in vitro* for prolonged periods (up to 28 to 30 days after HBV infection). We used confocal laser scanning microscopy to evaluate the cellular localization of HBcAg and HBsAg over the duration of infection.

**FIG 7:**
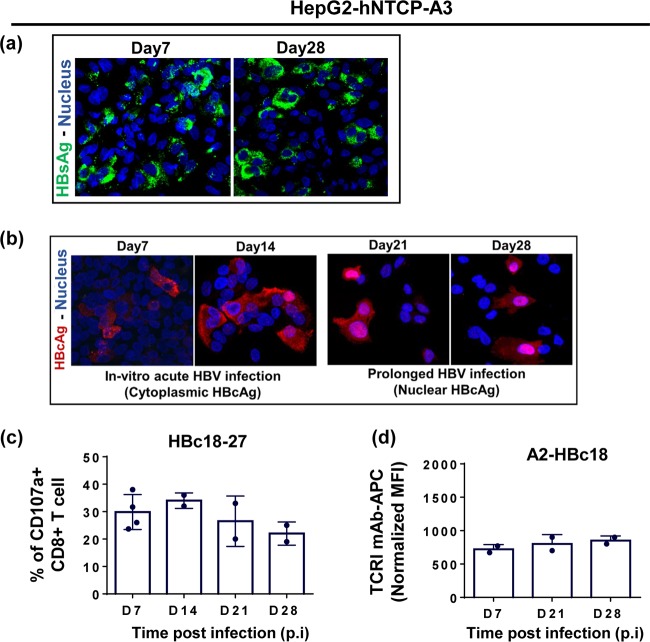
Cytosolic to nuclear relocalization of HBcAg does not alter HBc18-27 epitope presentation. (a) Cytoplasmic distribution of HBsAg at days 7 and 28 postinfection in HepG2-hNTCP-A3 cells using confocal laser scanning microscopy. HBsAg was stained in green using anti-HBs antibody while the nucleus was stained blue using DAPI. (b) HBcAg relocalization from cytosolic (days 7 and 14 postinfection) to more nuclear localization (21 and 28 days postinfection) during HBV infection in HepG2-hNTCP-A3 cells using confocal laser scanning microscopy. HBV-infected HepG2-hNTCP-A3 cells were stained with anti-HBc antibody (red), and DAPI (blue) was used to stain the nucleus. (c) Ability of CD8^+^ T cells specific for HBc18-27 to recognize HepG2-hNTCP-A3 cells infected with HBV for the indicated time. Shown is the frequency of the CD8^+^ T cells positive for CD107a among total CD8^+^ T cells upon coculture with HepG2-hNTCP-A3 targets for 5 h at an E/T ratio of 1:2. Data are shown as means ± standard deviations of at least two independent experiments. (d) Direct quantification of A2-HBc18 complexes on the surface of HBV-infected HepG2-hNTCP-A3 cells kept in culture for a prolonged duration of infection. Bars represent the MFI values of A2-HBc18 on HBV-infected HepG2-hNTCP-A3 cells normalized to the value in uninfected cells at each time postinfection. MFI values were measured using an ImageStream analyzer.

HBsAg showed a diffuse cytoplasmic and/or membranous pattern irrespective of the length of infection (Fig. 7a). In contrast, HBcAg displayed a predominantly cytoplasmic distribution during early phases of infection (days 7 and 14), while during prolonged infection (days 21 and 28), its localization increased in the nucleus. This variable intracellular localization has also been observed in the liver of patients with chronic HBV infection and has been hypothesized to regulate HBV-specific T cell recognition (Fig. 7b) (21, 22). Thus, we determined whether the final localization of core antigen (from the cytoplasm to nucleus) in HBV-infected HepG2-hNTCP-A3 cells (Fig. 7b) might alter the efficiency of HBV antigen presentation. No difference in the quantities of A2-HBc18 complexes or the activation levels of HBc18-27-specific CD8^+^ T cells was observed at days 7, 14, 21, and 28 after HBV infection in HepG2-hNTCP-A3 cells (Fig. 7c and d). Thus, these data show that the final different intracellular localizations of core antigen do not alter the processing and presentation of the HBc18-27 epitope.

## DISCUSSION

The HBV-infected liver contains a mosaic of hepatocytes expressing HBV antigens in different quantities and localizations (23–25) and from different sources (cccDNA or HBV DNA integration) (26). Here, we analyzed, to our knowledge for the first time, not the distribution of HBV proteins but that of HLA class I/HBV peptide complexes within the hepatic parenchyma. Even though our analysis is restricted to only two anti-HBe^+^ CHB patients, we directly observed that HLA class I/HBV epitopes may be not equally distributed in the liver but, on the contrary, may be preferentially present in distinct and sometimes mutually exclusive hepatic zones.

We then analyzed the possible causes of this target heterogeneity in HBV-infected PHH and hepatoma cell lines either infected with HBV (HepG2-hNTCP-A3) or with HBV DNA integration (HepG2.2.15, HepG2-Env, and PLC/PRF5-A2^+^ cells). By using HBV-specific CD8^+^ T cells and antibodies specific to HLA class I/HBV peptide complexes, we demonstrated that presentation efficiency of different HLA class I-restricted HBV epitopes is modulated by the presence of IFN-γ and by the level of production of newly translated antigens.

These findings might have important consequences for the design of immunotherapies targeting HBV chronically infected liver since CD8^+^ T cells of different HBV epitope specificities would not have identical capacities to recognize the heterogeneous population of HBV-infected hepatocytes.

Furthermore, by comparing the ability of cells in expressing HBV antigens from infection (PHH and HepG2-hNTCP-A3 cells) and from integration (HepG2.2.15, HepG2-Env, and PLC/PRF5-A2^+^ cells), we showed that the HLA-A*02:01 immunodominant HBs183-91 envelope epitope (37) was presented more efficiently in targets with HBV DNA integration than in HBV-infected HepG2-hNTCP-A3 cells. Future studies need to be performed to understand whether the differences can be generalized to normal HBV-infected hepatocytes with HBV DNA integration

These findings depict a scenario according to which hepatocytes with HBV DNA integration could act as a decoy for HBV-specific CD8^+^ T cells, sparing HBV-infected hepatocytes from recognition. Clearly, these data need to be confirmed for other HBV epitopes restricted by different HLA class I molecules and in a larger population of CHB patients. Nevertheless, if HBV DNA integration represents the major and constant source of newly synthetized HBsAg, particularly in anti-HBe^+^ CHB patients (26), the possibility that envelope-specific CD8^+^ T cells in anti-HBe^+^ patients would preferentially target hepatocytes with HBV DNA integration and not with productive HBV infection appears possible.

On the other hand, the efficient and consistent presentation of viral epitopes derived from HBV DNA integration might open a therapeutic opportunity for the treatment of HBV-related hepatocellular carcinoma. We have already shown that T cells engineered with HBV-specific T cell receptors can target HBV antigens in HCC cells with HBV DNA integration (38). Understanding the efficiency of HBV epitope presentation in HBV-infected primary hepatocytes or tumor cells carrying HBV DNA integration will allow the generation of engineered CD8^+^ T cells with T cell receptors specific for epitopes mainly produced by HCC cells and not by HBV-infected hepatocytes.

There are several limitations in this study. First, we defined the differential distribution of two HBV epitopes in the biopsy specimens of only two CHB patients. Our TCR-like antibodies detect HBV epitopes originated from HBV genotype D patients, and as such the pool of HLA-A*02:01^+^ CHB patients showing positive staining with both TCR-like antibodies was reduced to only three CHB patients. Furthermore, in only two patients did the consecutive sections stained with the two different HBV epitopes have an identical morphology that allows us to compare the topological localization of two different HBV epitopes. In addition, the differential distribution of core and envelope epitopes was detected in CHB patients that were anti-HBe^+^. Since the quantity of HBsAg produced from HBV DNA integration has been shown to be predominant in this CHB patient population (26), future studies will be necessary to define whether the mosaic distribution of different HBV epitopes can be generalized to other CHB patient populations.

In addition, the TCR-like antibodies used here limited our analysis to the HLA-A*02:01-restricted HBc18-27 and HBs183-91 epitopes. Although these epitopes are important in HLA-A*02:01^+^ subjects (31), they might not be representative of other nucleocapsid or envelope epitopes restricted by different HLA class I molecules.

For example, a core-derived HBV epitope restricted by HLA-A68w and HLA-A31 requires the activation of the immunoproteasome (20). This clearly differs from our results indicating that the HBc18-27 epitope remains unchanged upon treatment with IFN-γ. Similarly, we doubt that all envelope-derived epitopes restricted by different HLA class I epitopes will follow the same pattern of presentation as the HBs183-191 epitope reported here. For example, the clear dominance of the HLA-Cw0801 epitope HBs178-185 detected in Asian populations (39) might suggest that its generation is mediated by constitutive proteasome activity similar to what is observed in the immunodominant HLA-A*02:01/HBc18-27.

Nevertheless, the differences in the presentation efficiencies of the two HBV epitopes described here suggest that during natural infection, HBV-specific CD8^+^ T cells of different specificities might target selected HBV antigen-expressing hepatocytes with different efficacies within an infected liver. These findings called for a deeper understanding of the HBV epitope hierarchy of presentation across different HLA class I backgrounds in order to design immunological strategies to control chronic HBV infection or HBV-related HCC in patients.

## MATERIALS AND METHODS

### Cell lines.

Table 2 lists the biological features of cell lines used in the experimental settings.

**TABLE 2:** List of cell lines used in experimental systems

Cell line	Description	Reference
HepG2	HLA-A*02:01^+^, HCC line with no HBV DNA integration	
HepG2-hNTCP-A3 (A3 clone)	HepG2 cells overexpressing HBV entry receptor (hNTCP)	14
HepG2.2.15	HepG2 cells with full HBV genome integration producing virions	10
HepG2-Env	HepG2 cells transduced with HBV Env	35
PLC/PRF5-HLA-A*02:01	Natural HCC line with partial HBV surface antigen DNA integration; transduced with the HLA-A*02:01 molecule	36
EBV core	HLA-A*02:01^+^, EBV immortalized B cell lines transduced with HBV core DNA	8
HepAD38	HepG2 cells with full HBV genome integration producing virions	40

Briefly, the human liver cancer line HepG2 (ATCC), HepG2-hNTCP-A3 (HepG2 cells transduced with human NTCP) (14), and HepG2.2.15 (HepG2 cells transduced with full HBV genome) (10) cells were maintained in Dulbecco’s modified Eagle’s medium (DMEM) supplemented with 10% heat-inactivated fetal bovine serum (FBS), 100 U/ml of penicillin, 100 μg/ml of streptomycin, and GlutaMAX (Invitrogen, Carlsbad, CA). HepG2-hNTCP-A3 and HepG2.2.15 cells were selected using 5 μg/ml of puromycin (BD Biosciences, San Jose, CA) and 200 μg/ml Geneticin (G418 disulfate salt) (Sigma-Aldrich, St. Louis, MO), respectively.

HepG2-Env (35), PLC/PRF5/A2^+^ (PLC/PRF5 [36] transduced with HLA-A*02:01), and Epstein-Barr virus (EBV) core cells (EBV-transformed B lymphoblastoid cell line transduced with full HBV core protein) (8) were maintained in RPMI 1640 medium supplemented with 10% heat-inactivated FBS, 20 mM HEPES, 0.5 mM sodium pyruvate, 100 U/ml penicillin, 100 μg/ml streptomycin, minimal essential medium (MEM) amino acids, GlutaMAX, and MEM nonessential amino acids (Invitrogen, Carlsbad, CA). HepG2-Env and PLC/PRF5/A2^+^ cells were selected using 5 μg/ml puromycin. EBV core cells were selected using 250 μg/ml hygromycin (Sigma-Aldrich).

HepAD38 cells, used for virus particle production, were cultured in DMEM with 10% tetracycline-free FBS, 100 U/ml penicillin-streptomycin, 2 mM L-glutamine, and 0.4 ug/ml doxycycline (40).

### PHH culture.

Fresh HLA-A*0201^+^ primary human hepatocytes (PHH) were obtained from a humanized FRG mouse model (Invitrocue, Singapore). PHH were maintained at a distinct density according to the manufacturer’s protocol in Hepacur medium (Invitrocue, Singapore) containing 2% dimethyl sulfoxide (DMSO) in 37°C with 5% CO_2_ all through the experiment.

### HBV virus production.

Briefly, to induce virus particle production in HepAD38 (HBV genotype D), doxycycline was removed from the medium, fresh medium was replaced, and after 20 days, HBV DNA titers were measured from the supernatant by quantitative PCR (qPCR), according to manufacturer's instructions, using a Qiagen HBV Artus PCR kit (Qiagen). Virus particles were subsequently concentrated using a commercial polyethylene glycol (PEG) precipitation kit (Abcam) according to manufacturer's protocol, which resulted in approximately 50- to 100-fold concentrations of virus stock.

### HBV infection.

HepG2-hNTCP-A3 cells at 70% confluency and PHH at day 1 postseeding were inoculated with approximately 50- to 100-fold-concentrated supernatant of HepAD38 as an HBV inoculum (genotype D) at a multiplicity of infection of 3,000 GEV/cell in 4% PEG (Sigma-Aldrich) medium for 24 h at 37°C with 5% CO_2_. Inoculum was removed subsequently, and cells were washed with 1× phosphate-buffered saline (PBS) three times. Infection efficiency was quantified at 12 and 18 h and at days 1, 3, and 7 for both HepG2-hNTCP-A3 cells and PHH after removal of inoculum, referred to as time postinfection.

### Coculture experiment of HBV-specific CD8^+^ T cells with targets.

Two HLA-A*02:01 CD8^+^ T cell lines specific to HBV epitopes core 18-27 (HBc18-27) and S183-91 (HBs183-91) were generated from HLA-A02:01^+^ patients with acute hepatitis infection and maintained *in vitro* as described previously (41).

The activation of HBV-specific CD8^+^ T cells was tested by measuring degranulation (CD107a) and cytokine production (IFN-γ and TNF-α) through surface and intracellular staining, respectively. Briefly, CD8^+^ T cells were incubated with different cell lines for 5 h in the presence of brefeldin A (BFA) (10 μg/ml) and CD107a-fluorescein isothiocyanate (FITC) (BD Biosciences) at an effector/target (E/T) ratio of 1:2 at 37°C with 5% CO_2_. After cells were washed, they were subjected to surface and intracellular staining.

Both CD8^+^ T cell clones were activated by HepG2.2.15 (HepG2 cells with full HBV genome integration) (10), demonstrating their ability to recognize HBV epitopes produced from endogenously synthetized HBV antigens (data not shown). Analysis of functional affinity of the CD8^+^ T cell clones showed that HBc18-27- and HBs183-91-specific CD8^+^ T cells can recognize target cells (HLA-A*02:01^+^ EBV immortalized B cells) pulsed with peptide concentrations as low as 1 to 10 pM.

### Surface and intracellular staining.

**(i) HBV-specific CD8^+^ T cell activation.** After 5 h of incubation with CD8^+^ T cell clones in the presence of CD107a and BFA (as described earlier), cells were stained with anti-CD8 V500 (BioLegend) for 30 min at 4°C, followed by a standard intracellular staining protocol. Briefly, cells were fixed and permeabilized for 30 min at 4°C (CytoFix/CytoPerm; BD Biosciences), followed by staining with mouse anti-human IFN-γ phycoerythrin (PE)-Cy7 (BioLegend) and mouse anti-human TNF-α–PE (BD Biosceinces) for 30 min at 4°C. Finally, cells were fixed in 1% paraformaldehyde (PFA) in 1× PBS; cell acquisition was performed using a BD LSR-II flow cytometer, and data were analyzed in FACSDiva software.

**(ii) HBV infection efficiency.** HBV-infected or uninfected cells at different times postinfection were fixed and permeabilized for 30 min at 4°C (CytoFix/Cytoperm; BD Biosciences). Cells were then stained with primary antibodies, rabbit HBcAg (ThermoFisher Scientific) and mouse HBsAg (RayBiotech), for 30 min at 4°C. This was followed by 30 min of staining using the secondary antibodies goat anti-rabbit-CF55 (Sigma-Aldrich) and goat anti-mouse-allophycocyanin (APC) (Invitrogen) at 4°C. Cells were then fixed in 1% PFA in 1× PBS; acquisition was done using a BD LSR-II flow cytometer, and data were analyzed with FACSDiva software.

### HBV-epitope/HLA-A*02:01 complex quantification (TCR-like mAb staining).

Two antibodies specific to HBc18-27 and HBs183-91/HLA-A*02:01 complexes were used for staining PHH, hepatocyte-like cell lines, and liver biopsy specimens. Their production and specificity were described previously (17). Since these antibodies, similar to T cell receptors of T cells, recognize the complexes HBV peptide/HLA-A*02:01 molecules, we defined them as TCR-like antibodies.

**(i) *In vitro* HBV infection system.**

***(a)* Flow cytometry analysis.** Infected or uninfected cells were stained with an Aqua Live/Dead fixable dead cell stain kit (Invitrogen) for 10 min at room temperature (RT), followed by staining with TCR-like antibodies for 1 h. Cells were then stained with goat anti-mouse-APC secondary antibody (Invitrogen). Cells were then subjected to an APC Faser amplification kit (Miltenyi Biotech). This step was followed with intracellular staining for HBcAg (as described earlier). APC mean fluorescence intensity (MFI) in cells positive for HBcAg was analyzed using a BD LSR-II flow cytometer. Data analysis was done using Kaluza software (Beckman Coulter).

***(b)* ImageStream analysis.** Infected or uninfected cells were stained with TCR-like antibodies for 1 h, followed by staining with goat anti-mouse-APC secondary antibody for 30 min. APC signal was amplified as described earlier. The MFI of APC was then analyzed using an ImageStream analyzer. Images were analyzed using IDEAS, version 4.0, software.

**(ii) Liver biopsy specimen staining.** Briefly, human liver biopsy samples from 8 HLA-A*02:01 patients as described in Table 1 were kept frozen in optical cutting temperature (OCT) medium (VWR Chemicals) before staining. Tissues were then sectioned (5 μm) and fixed in acetone for 30 min, followed by air drying for 10 min. Samples were then washed with 1× PBS, followed by two-step blocking with a dual-endogenous enzyme block (Dako/Agilent Technologies) and 2% BSA in 1× PBS at RT for 10 and 30 min. Tissues were incubated with the above-mentioned TCR-like antibodies overnight at 4°C. This step was followed by anti-mouse secondary antibody staining using an EnVision+ System with horseradish peroxidase (HRP)-labelled polymer (Dako/Agilent Technologies) for 30 min at RT. Tissues were then subjected to a tyramide staining-Alexa Fluor 647 (Thermo Fisher Scientific) amplification kit, followed by costaining with mouse anti-human cytokeratin 18-FITC (Miltenyi Biotech) and nucleus staining with 4′,6′-diamidino-2-phenylindole (DAPI). Whole-tissue scanning and fluorescence microscopy were performed on an automated scanning workstation (TissueFAXS; Tissue Gnostics).

### Viral antigen staining in liver biopsy specimens.

Tissue biopsy specimens kept in OCT medium (as mentioned previously) were sectioned (5-μm) and washed with 1× PBS. Slides were then blocked and permeabilized using 3% mouse serum, 1% BSA, and 0.25% saponin in 1× PBS. This was followed with staining of tissue sections with the primary antibody goat anti-HBsAg (Abcam) overnight at 4°C. Tissue sections were then subjected to staining with secondary antibody, rabbit anti-goat-APC coupled with cytokeratin 18-FITC-conjugated antibody (Miltenyi Biotech). Cells were then stained with DAPI for nucleus staining. Images were captured using whole-tissue scanning and fluorescence microscopy on an automated scanning workstation (TissueFAXS; Tissue Gnostics).

### Immunofluorescence staining.

HBV-infected HepG2-hNTCP-A3 cells were seeded on coverslips at a density of 2 × 10^5^ cells. At indicated days postinfection, cells were washed with 1× PBS and fixed with 4% PFA for 10 min. Upon permeabilization, cells were stained using primary antibodies rabbit anti-HBcAg (Abcam) and mouse anti-HBsAg (RayBiotech) followed by secondary antibodies goat anti-rabbit CF633 (Sigma-Aldrich) and goat anti-mouse Alexa Fluor-488 (Invitrogen). Cell nuclei were then stained with DAPI, and images were acquired using Carl Zeiss confocal laser scanning upright microscope.

### NanoString gene expression analysis.

Targets with HBV DNA integration as well as HBV-infected or uninfected cells were lysed in RLT lysis buffer (Qiagen) (supplemented with 2-mercaptoethanol at 1:100) according to NanoString Technologies guidelines. Lysate of at least 20,000 cells was analyzed using the customized nCounter GX human Immunology kit coupled with probes specific to HBV viral RNA. A probe set specific to HBV viral RNA was designed according to NanoString nCounter Technology guidelines (NanoString Technologies, Seattle, WA) to specific regions on the HBV genome, as shown in Fig. 2b.

The background detection and normalization of data were done using n-Solver analysis software, version 3.0, based on the geometric mean of the supplied positive and negative controls and the housekeeping gene panel.

### Statistics.

Statistical significance was evaluated with a two-tailed *t* test and, where appropriate, one-way or two-way analysis of variance (ANOVA) with Tukey’s or Dunnett’s multiple-comparison test using the data analysis software Prism, version 6. Only *P* values and adjusted *P* values (ANOVA) of less than 0.05 were considered significant and displayed in the figures.

### Ethics statement.

Written informed consent was obtained prior to collection of liver samples. The study was approved by Barts and the London NHS Trust local ethics review board and the National Research Ethics Service (NRES) Committee London (Research Ethics Committee reference 10/H0715/39).
